# Korean Learners’ Acquisition of Mandarin Disyllabic Tone Sequences Across Proficiency Levels

**DOI:** 10.3390/brainsci16010021

**Published:** 2025-12-24

**Authors:** Yuping Fu, Yong-cheol Lee, Yanyang Zheng

**Affiliations:** 1 School of Foreign Languages, Hainan Tropical Ocean University, Sanya 572000, China; 2 Department of English Language & Literature, Hannam University, Daejeon 34430, Republic of Korea; soongdora@hnu.kr; 3 School of Foreign Languages, Nanyang Institute of Technology, Nanyang 473000, China; vidazheng@126.com

**Keywords:** Korean learners, non-tonal language, Mandarin tones, prosodic mapping, tone sequence production, error patterns, motor theory, neurolinguistic constraints

## Abstract

**Background**: Although tone acquisition is one of the most challenging aspects for adult second language (L2) learners, research remains limited on how learners from non-tonal first language (L1) backgrounds develop across proficiency levels. The current study examined Mandarin disyllabic tone sequences produced by learners at three proficiency levels. **Methods**: This study recorded the Mandarin tone production of beginner, intermediate, and advanced Korean learners and evaluated their accuracy and error patterns to determine whether similarities between L1 and L2 prosodic systems affect tone sequence difficulty. **Results**: Across groups, tone sequence rankings were consistent, differing mainly in accuracy rates. Learners showed an advantage in producing sequences aligned with Korean tonal patterns, such as T1–T1 and T3–T1, which were the easiest to produce. In contrast, sequences without Korean counterparts, particularly those ending in T2, remained the most difficult at all proficiency levels. **Conclusions**: Neurolinguistic evidence suggests that tones lacking L1 motor representations are disadvantaged by limited motor templates and weaker auditory coding, which together account for persistent difficulty with T2 sequences. Interestingly, T2 in word-initial position improved with experience, as increased exposure and practice helped learners form new sensorimotor routines supported by strengthened auditory–motor coupling. Over time, such experience-dependent neural reorganization enables more precise execution of rising F0 movements when tones occur at the beginning of a sequence, whereas carry-over interference from preceding tones continues to hinder accuracy in word-final position. This study provides insight into how sensorimotor and auditory systems interact in L2 tone learning, offering a neurocognitive framework for understanding prosodic transfer.

## 1. Introduction

Acquiring tones has been found to be one of the most difficult aspects of prosody and poses tremendous challenges to adult second language (L2) learners, particularly those whose first language (L1) is non-tonal [1,2,3,4,5,6,7,8,9,10,11]. This is because tonal and non-tonal languages contain very different prosodic structures. Unlike tonal languages, Seoul Korean (Korean, hereafter) fundamentally uses pitch (also known as fundamental frequency, F0) for intonational purposes. A falling pitch contour (we define F0 movement as the local, dynamic changes in pitch over a short stretch of speech (e.g., rising, falling, or dipping patterns within a syllable), and F0 contour as the overall pitch shape spanning the entire syllable or phrase) often refers to a statement in a sentence-final position and a rising pitch contour often signals a question. However, pitch is modulated over each syllable in tonal languages like Mandarin Chinese (Mandarin, hereafter), and identical syllables with different pitch shapes have distinct lexical meanings. For instance, the syllable da with a high-level tone means build but with a rising tone means reach.

This study investigates L2 acquisition of Mandarin disyllabic tone sequences by non-tonal Korean learners with three different proficiency levels. Specifically, participants will produce Mandarin disyllabic tone sequences, and their tone productions will be evaluated by Mandarin native speakers. Prior to presenting our research questions, the lexical tones of Mandarin and the prosodic structures of Korean first need to be introduced as background information for this study. Next, a review of relevant literature on the production and perception of Mandarin tones will be provided. Finally, the research questions that bridge the gaps identified in previous studies will be presented.

### 1.1. Lexical Tones in Mandarin

There are four lexical tones in Mandarin, as shown in Table 1. Tone 1 is a high-level tone, tone 2 a mid-rising tone, tone 3 a low-dipping tone, and tone 4 a high-falling tone. Relative height of pitch in each tone is labeled with numbers 1–5 with 5 indicating the highest pitch and 1 the lowest [12] (p. 26). As Table 1 illustrates, the homophonous segment da with four differing tones represents four different words. For learners of Mandarin, it is therefore essential to understand both how tones are actually produced and how they distinguish word meanings. The four tones constitute a systematic classification of pitch contours that link tonal shape to lexical meaning. For example, to express “big” (da, T4), a learner must know that the correct tone is high-falling (T4). A Mandarin speaker’s linguistic knowledge ensures that producing this syllable with Tone 1 instead would result in a misunderstanding. When the brain instructs the speech organs to produce the high-falling Tone 4, the relevant muscles adjust the vocal folds so that their vibration frequency decreases from a high to a low pitch range appropriate for that tone [13].

Based on Duanmu’s proposal [14] and Yip’s [15] assumption of underlying phonological tone targets in Mandarin, the tonal representations of each tone are shown in (1). T1 is associated with a high-level pitch (55). T2 and T4 are represented by mid-high (35) and high-low (51) contours, respectively. Full T3 has a mid-low-high (214) representation and half T3 has a low-falling or low-level pitch contour. But the exact nature of T3 remains unclear. When preceding T1, T2 and T4, half T3 could be realized as 21, 22, or 11 [16]. Nevertheless, the difference between the full T3 and the half T3 is that in the half T3, the rising part (14) is missing.

(1) Mandarin tonal representation (H: high; M: mid; L: low)


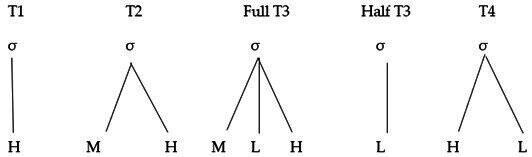


Using the four lexical tones, 16 tone sequences can be combined for disyllabic words, as in Table 2. The row displays a target tone in Syllable 1 of the disyllabic word and the column indicates a target tone in Syllable 2.

### 1.2. Prosodic Structures in Seoul Korean

In Korean, pitch does not directly encode lexical meaning through tonal contrasts but instead functions as a phonetic correlate of segmental contrasts [17,18,19]. Jun’s [18] autosegmental–metrical framework of intonational phonology proposes two prosodic units above the Word (W): Intonational Phrase (IP) and Accentual Phrase (AP). Within an AP, Korean has two prosodic templates (HH-LH and LH-LH; H for high and L for Low) and the AP-initial tone is determined by the laryngeal feature of an AP-initial segment. When the AP-initial segment is aspirated or tense, the AP starts with a H tone, but otherwise, a L tone. LHLH or HHLH is the full realization of an AP when the number of syllables with an AP is four or more, but with fewer syllables, the medial L or H or both is unspecified, resulting in 14 different tonal patterns, as follows: LH, LHH, LLH, LHLH, HH, HLH, HHLH, LL, HL, LHL, HHL, HLL, LHLL, HHLL [20]. Table 3 summarizes the tonal patterns of 16 disyllabic tone sequences in Mandarin and their corresponding tonal realizations in Korean. It should be noted that the T4–T1 sequence in Mandarin appears to correspond to HLH in Korean prosody. However, Korean tonal patterns beginning with HL, as listed in Table 3, occur only in sentence-final positions. As such, these HL-initial patterns are not relevant to the present study. Because HL cannot occur in word-initial position in Korean, Korean learners of Mandarin may experience difficulty producing this tone sequence at the beginning of a word. They are likely to overcome this difficulty with experience as their proficiency improves, given that HL can occur sentence-finally in declarative sentences.

In the present study, tonal sequences are compared across Mandarin and Korean at the level of surface pitch movements across adjacent syllables, rather than at the level of syllable–tone association. Mandarin lexical tones are phonologically associated with syllables, but may surface with complex pitch movements due to contour tones and sandhi processes. In contrast, Korean pitch patterns are realized as sequences of tonal targets distributed across syllables, with at most one tonal target per syllable. Accordingly, when a tonal melody such as HLH is described as being common to both languages, this refers to similarity in the linear ordering of pitch targets, not to identical syllable–tone association. For example, HLH corresponds to HL-H in Mandarin but H-L-H in Korean. Our intention is not to claim equivalence in syllable–tone association, but rather similarity in surface pitch sequencing.

### 1.3. Literature Review

When acquiring tone languages, phonetically similar tone pairs are known to be more challenging than phonetically dissimilar ones [21,22,23]. Regardless of their L1 backgrounds, L2 learners tend to make similar errors when producing these similar sequences [10,24,25]. In Mandarin, sequences such as T1–T2, T4–T1, T2–T2, T2–T3, and T4–T4 often cause confusion. The difficulty with T1–T2 arises from their shared phonetic feature of a high pitch at the final offset, while T4–T1 is challenging because both tones start with a high pitch. In contrast, tone pairs like T1–T3, T3–T1, and T3–T4, which have dissimilar phonetic features in their initial onset and final offset pitch points, are relatively easier to acquire. These tonal confusions, driven by the phonetic similarities of tone sequences, appear to be language independent [21].

Previous studies on L2 tone acquisition in disyllables have yielded conflicting results, possibly due to differences in learners’ L1 backgrounds. Cantonese learners of Mandarin struggled with T2–T3 [26], while this sequence was among the easiest for Japanese and Korean learners [10]. Japanese learners produced T3–T1 relatively accurately [10], whereas English speakers found this sequence challenging [27]. The mapping between L1 prosodic patterns and L2 tone pairs may explain such tonal confusion across different L1 groups [25,28]. This suggests that L2 learners from different L1 backgrounds may produce L2 tones in ways influenced by their L1 prosody. This perspective underscores the importance of prosody as an area where L1s differ, indicating that the availability of certain tone contours in the L1 prosodic system may influence which L2 tone sequences are easier or more challenging to produce.

To sum up, prior research on L2 tone acquisition has revealed two key insights: (1) tone pairs with similar phonetic features tend to be more difficult to acquire than those with dissimilar features, and (2) L1 prosodic systems can either facilitate or hinder L2 tone acquisition. Applying these insights to Korean learners, we can anticipate varying levels of difficulty across different Mandarin tone pairs, such as T1–T1 and T3–T1. The T1–T4 sequence is expected to pose fewer challenges, as it aligns with (or approximates) patterns found in the Korean prosodic system, providing Korean learners with a relative advantage. In contrast, T1–T2, which lacks a comparable counterpart in Korean, is likely to be more difficult to acquire. Although there is no exact one-to-one correspondence between Mandarin tone sequences and Korean prosodic patterns, Mandarin tone sequences that are prosodically similar to Korean AP patterns tend to be easier to acquire due to L1 prosodic transfer effects. Studies by Francis et al. [28] and Yang [25] suggest that the degree of mapping between L1 prosodic patterns and L2 tone pairs—whether similar or dissimilar—shapes acquisition patterns. When L1 and L2 share prosodic similarities, learners may experience a facilitative effect, whereas dissimilar patterns can lead to greater learning difficulty. This aligns with Gampe et al. [29], who argue that linguistic similarities between two languages play a significant role in foreign language acquisition.

By analyzing the similarities and differences between Korean tonal patterns and Mandarin tone sequences, we can predict which Mandarin tone pairs Korean learners are likely to find easier or more challenging to produce, as outlined below.

(1) Producing tone sequences like T1–T1 (H-H), T3–T1 (LH-H), and T1–T4 (H-HL) would be relatively easier, as these are similar to tonal patterns found in Korean.
(2) Producing tone pairs involving T2 (MH) in any syllable position and T4 (HL) in word-initial positions would be more challenging, as there are no comparable patterns in Korean prosody.
(3) The difficulty of producing other sequences may fall between these extremes.

Beyond phonetic similarity and prosodic transfer, recent research emphasizes neurolinguistic constraints on tone learning. From a motor theory perspective, speech production and perception are grounded not only in acoustic mapping but also in the neural simulation of motor gestures [30,31]. Korean learners can draw on existing motor routines for intonational patterns similar to Mandarin sequences such as T1–T1 and T3–T1, making these tones easier to reproduce. However, Mandarin T2 (the mid-rising tone) has no motor counterpart in the Korean prosodic system, leading to unstable motor simulation and persistent difficulty even at advanced proficiency levels.

ERP studies provide converging evidence: the mismatch negativity (MMN) component, an automatic neural response reflecting pre-attentive auditory discrimination, is often attenuated or delayed in non-tonal L1 learners due to the lack of auditory salience when processing unfamiliar rising tones such as T2 [32]. fMRI studies further reveal weakened connectivity between auditory regions and motor-related areas during unfamiliar tone processing [33], and hence learners from non-tonal language backgrounds must develop novel processes for perceiving lexical tones [8]. These findings suggest that tones without equivalents in the L1 motor repertoire are particularly disadvantaged—lacking both motoric templates and automatic auditory encoding. This dual constraint helps explain why Mandarin T2 may remain especially challenging for Korean learners across proficiency levels.

This study investigated 16 tone combinations in Mandarin disyllabic words, focusing on Korean learners of Mandarin across three proficiency levels: beginner, intermediate, and advanced. The emphasis on disyllabic words is crucial, as approximately 70% of Mandarin words are disyllabic [34]. Previous research on L2 Mandarin tone production has primarily centered on English learners [1,2,4,10,27,35,36,37,38,39] but recent studies [10,25,26,36,40,41,42,43,44] have expanded to include speakers of other languages, such as Cantonese, French, Indonesian, Japanese, Korean, Thai and Yoruba. However, these studies, including two studies examining Korean learners [10,43], have not adequately considered varying proficiency levels, focusing exclusively on intermediate learners. By examining Korean learners across different proficiency levels, this study seeks to better understand how Korean tonal patterns—whether similar or dissimilar to those in Mandarin—affect the production of L2 Mandarin tones. This approach will enhance our understanding of how L1 prosodic systems influence L2 tone sequence production and how learners’ accuracy in tone sequence production improves with increased proficiency. Specifically, we will answer the following three research questions:

First, is it relatively easier for Korean learners to produce tone sequences like T1–T1 (H–H), T3–T1 (LH–H), and T1–T4 (H–HL), which are similar to tonal patterns found in Korean?

Second, is it more challenging for Korean learners to produce tone pairs involving T2 (MH) in any syllable position and T4 (HL) in word-initial positions with no comparable patterns in Korean prosody?

Third, will the difficulty of producing other sequences fall between these extremes?

The methodology of this study will be detailed in the following section.

## 2. Materials and Methods

### 2.1. Participants

Previous studies examining Korean learners’ Mandarin tones have lacked detailed proficiency classifications. Zhang [10] classified learners with 0.5–1.5 years of experience as intermediate, while Hao [26] considered two years of experience as intermediate, and Wang et al. [45] classified two years of experience as advanced. Therefore, this study adopted a more objective approach by classifying learner groups based on their proficiency in Mandarin using the levels of Hanyu Shuiping Kaoshi (HSK), an official test of Mandarin proficiency for non-native speakers. It includes six levels. Level 1 and Level 2 are labeled as beginners, Level 3 and Level 4 as intermediates and Level 5 and Level 6 as advanced [46].

We recruited three groups of Korean learners from Cheongju and Seoul, Korea, and Beijing, China. The participants were distributed as follows: 14 from Cheongju, 12 from Hongik University, and 10 overseas Korean students from Beijing Language and Culture University. None had experience learning tonal languages except Mandarin. Group 1 had 12 beginner learners (average age: 21.83, SD: 3.04) with Mandarin learning experience ranging from three months to one year (average: 7.2 months). They were freshmen in their first semester of Mandarin learning. Their Chinese teacher administered a simulated HSK-2 test to assess their proficiency and classified them as beginners based on the results. Group 2 included 12 intermediate learners with HSK 4. Their average age was 25.17 (SD: 5.31) and their experiences learning Mandarin are about two years, averaging 25.67 months. Group 3 comprised 12 advanced learners with HSK 6 (average age: 29.17, SD: 8.27), who had been learning Mandarin for about 4 years (average: 53.3 months). We also recruited 12 native Mandarin speakers as a control group. All participants were undergraduate and graduate students from Hainan Tropical Ocean University. They were raised in northern China and received their primary and secondary education there (mean age = 24.7 years, SD = 2.57).

### 2.2. Stimuli

With equal distribution of seven basic vowel phonemes, we formulated 16 tone sequences within each position of disyllabic words, totaling 112 combinations. Participants produced two different words for each pair, resulting in 224 words. These words were sourced from the General Outline of the Chinese Vocabulary Levels and Graded Chinese Characters (1992) and 5000 Graded Vocabulary for HSK Outline (2015), with four exceptions. Low-frequency words (e.g., cí.gēn ‘word root’) had to be included as selecting only high-frequency words from the two sources was challenging. Criteria for selecting morphemes primarily followed Zhang’s [10] guidelines. This involved prioritizing content words, minimizing word-initial obstruents, maximizing sonorants, favoring codaless syllables, and potentially using diphthongs.

To neutralize contextual tone effects [47], stimuli were embedded within carrier phrases, surrounded by neutral-tone particles ‘ge’ and ‘de’, as in (2).
(2)nàgekā.fēide
那个咖啡的
Thatcoffee‘s

This approach ensured consistent neutral-tone effects across all stimuli and minimized disruptions from sentence-final intonation.

### 2.3. Recording Procedure

Recordings took place in a sound-proof booth at Cheongju University and in quiet rooms at Hongik University, Beijing Language and Culture University and Hainan Tropical Ocean University. Participants sat comfortably before a laptop during the recordings. Using Praat [48], recordings were made at 44.1 kHz via a Sennheiser microphone headset and Lenovo ThinkPad laptop. Before the actual recording, the experimenter instructed participants to read naturally without emotional emphasis. Participants noted the experimenter’s gestures for re-reading in case of errors. 224 carrier phrases with hanzi, pinyin and tones were split into three blocks (75, 75, and 74 phrases) and displayed using PowerPoint slides. Participants practiced three samples before producing 448 target phrases (224 per round), pausing between blocks. The order of presenting stimuli was randomized for each participant. Each recording session lasted 40–50 min, yielding 16,128 speech samples (224 phrases × 36 participants × 2 rounds) by Mandarin learners and 5376 ones (224 phrases × 12 participants × 2 rounds) by native Mandarin speakers. Praat was used to segment each phrase for evaluation procedures.

### 2.4. Accuracy Rating

We recruited three native Mandarin raters to evaluate Korean learners’ accuracy in the production of tone sequences, a recognized method for assessing L2 production [8,26].

#### 2.4.1. Raters

Three raters (average age: 45) were selected based on specific criteria. They grew up in North China, were language majors, and had been language teachers for over 11 years at a university in Hainan Province. They had verified proficiency in standard Mandarin with PSC levels of Grade 2 Level 1 (PSC score ≥ 88,) (PSC, short for Putonghua Shuiping Ceshi, is an oral test assessing native Chinese Mandarin proficiency; it comprises three levels (levels 1–3), each further divided into two grades: A and B; the highest level is Grade A Level One (97–100), while the lowest is Grade B Level Three (60–69)). None of them had a history of hearing, speech, or language difficulties.

#### 2.4.2. Analyses

Before rating the target stimuli, the raters practiced using the native productions of 32 disyllabic words. These words were similar to the test set but used different disyllabic words. This session aimed to familiarize the raters with the rating procedure. The stimuli for this session were recorded by a female native Mandarin speaker whose age was 42 and had a PSC score of 97. The raters accurately rated all practice stimuli.

Subsequently, the judges rated the tone productions from the three learner groups. They received answer sheets containing 224 stimuli in Pinyin without tonal diacritics. By listening to carrier phrases, they recorded their responses on these sheets and replayed ambiguous productions when necessary. The judges annotated tonal diacritics and marked corresponding tone sequences on the right (e.g., 1-1, 1-2) [8]. Across all proficiency groups, raters showed very high levels of agreement. In the beginner group, 96.56% of ratings were agreed and 3.44% were disagreed, indicating slightly greater variability at the lowest proficiency level. Agreement increased in the intermediate group, where 97.58% of ratings were agreed and 2.42% were disagreed, and remained comparably high in the advanced group, with 97.62% agreement and 2.38% disagreement. Overall, disagreement was minimal across all groups, and agreement rates consistently exceeded 96%, suggesting strong inter-rater consistency regardless of proficiency level. Any initial disagreements were resolved through collaborative discussion among the raters with pitch-track reviewing in Praat to ensure consistency, after which full consensus was reached, and all final ratings were agreed upon. While chance-corrected reliability measures such as Cohen’s κ can further enhance methodological transparency, the present study reports raw agreement rates because final ratings were established through collaborative resolution rather than fully independent coding. Nevertheless, the consistently high agreement rates indicate strong inter-rater consistency prior to consensus. In this study, the tone pair productions from 36 learners generated a total of 48,384 responses (3 raters × 224 stimuli × 2 rounds × 36 learners).

We calculated the accuracy rates of learners’ tone productions using two methods. First, we evaluated the production of 16 tone pairs. The production of a tone pair was considered correct only if all the syllables within the pair were pronounced accurately. Next, we assessed the accuracy of each tone within disyllabic words. A tone was marked as correct if the target tone in the pair was pronounced accurately, regardless of whether the other tone was incorrect. For example, in the T1–T4 pair, as long as the first tone was correctly pronounced as T1, it was marked as accurate, even if the second tone was mispronounced.

Using these methods, we conducted three analyses. The first analysis ranked the accuracy rates of the 16 tone pairs within each learner group to identify which pairs were easier or more difficult to produce. Second, we examined the error patterns in tone pair productions, comparing correct and incorrect responses to determine if the learners’ L1 prosodic systems influenced these patterns. The third analysis calculated the accuracy rates for each tone within disyllabic words to determine which tones were more challenging or easier to produce based on their position within a word.

We employed the lme4 package [49] in R (Version 4.0) [50] to perform a binary logistic regression analysis. The fixed effects included proficiency level (beginner, intermediate, advanced), tone sequence (16 sequences), and syllable position (1st and 2nd syllables). Random effects were learners (12 per group), rounds (1, 2), and words (224 total). The dependent variable was the perception score assigned by the raters (0: incorrect, 1: correct). To assess the significance of the fixed effects, we conducted a likelihood ratio test using the ANOVA function. Additionally, we performed post hoc multiple comparisons with the emmeans package [51] to identify significant differences across target sequences.

## 3. Results

### 3.1. An Overview of Pitch Contours

To generate pitch contours across language groups, we analyzed 16,128 phrases produced by the three learner groups and 5376 phrases produced by the control group, encompassing all 16 Mandarin disyllabic tone sequences. Each word in the target phrases was first manually annotated using ProsodyPro [52], after which the script automatically extracted F0 values at ten equidistant normalized time points within each labeled interval, which allows duration-independent comparison of F0 contours across conditions and speakers.

**Figure 1:**
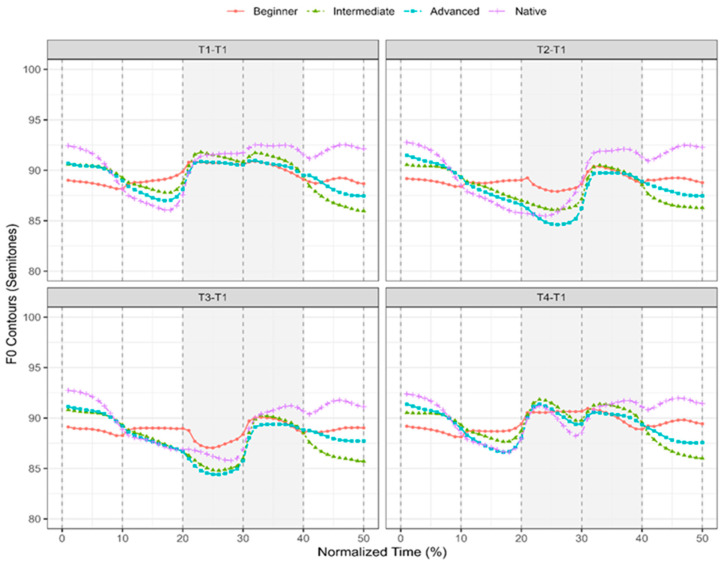
Time-normalized pitch contours (in semitones) for four Mandarin disyllabic tone sequences (T1–T1, T2–T1, T3–T1, T4–T1) across learner groups and native speakers.

Figure 1 displays time-normalized pitch contours averaged across 336 productions (12 speakers × 14 words × 2 rounds) for each language group. The shaded gray areas mark the target tone sequences, and the vertical dashed lines indicate word boundaries. The *x*-axis represents normalized time, and the *y*-axis displays F0 contours in semitones. The original F0 values in hertz were converted to semitones using the formula: semitone = 12 × log_2_(x). From Figure 1, we provide sample pitch contours to illustrate the general acoustic patterns of the tone sequences prior to the judgment test. In the T1–T1 sequence, learners across proficiency levels appear to produce the expected T1–T1 pattern. For the T2–T1 sequence, however, the initial tone (T2) tends to be realized with a noticeable dipping contour as T3. The T3–T1 pair shows a clearer realization of the dipping contour in the initial tone, suggesting the relatively accurate production in this tone pair. In the T4–T1 sequence, all learner groups except beginners show contours resembling the target T4–T1 pattern. Overall, the sample pitch contours suggest that the initial T2 remains particularly challenging even for advanced learners, whereas the initial T4 poses difficulty primarily at lower proficiency levels but improves with experience. These acoustic tendencies imply that in subsequent judgment tasks, T2–T1 may be identified as T3–T1, and beginner’s productions of T4–T1 may be perceived as T1–T1.

### 3.2. Overall Tendencies

**Figure 2:**
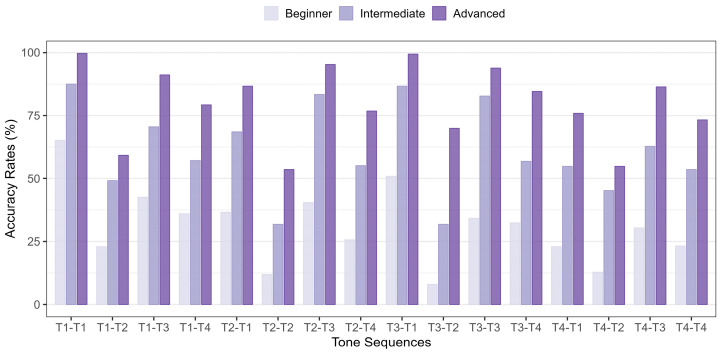
Accuracy rates for the 16 tone sequences produced by the three groups.

Figure 2 presents the accuracy rates of 16 tone sequences as produced by beginner, intermediate, and advanced Korean learners of Mandarin. The *x*-axis represents the tone sequences, while the *y*-axis indicates the accuracy rates assessed by the three judges. Two notable trends emerge from the results. First, accuracy rates generally increased as learners progressed from beginner to intermediate and then to advanced. Second, sequences containing Tone 2—particularly when it appears in the second syllable, except for T2−T3—consistently showed the lowest accuracy rates across all three learner groups.

Table 4 ranks the accuracy rates of 16 tone sequences across the three learner groups. Despite differences in proficiency, all groups exhibited similar patterns in the relative ease and difficulty of tone sequence production. For beginner learners, tone sequences with Tone 2 in the second syllable were the most challenging, particularly T3−T2 (8.0%) and T2−T2 (11.9%). In contrast, the easiest sequences were T1−T1 (65.2%), T3−T1 (50.9%), T1−T3 (42.6%), and T2−T3 (40.5%). A similar pattern was observed among intermediate learners, with T3−T2 (31.8%) and T2−T2 (31.8%) remaining the most difficult, while T1−T1 (87.5%), T3−T1 (86.6%), T2−T3 (83.3%), and T3−T3 (82.7%) had the highest accuracy rates. Advanced learners also struggled with T2−T2 (53.6%) and T4−T2 (54.8%). However, their accuracy rates for T1−T1 (99.7%) and T3−T1 (99.4%) were nearly perfect in producing these sequences.

Based on the results in Table 4, we conducted a binary logistic regression analysis to examine whether the production of the 16 sequences differed across the three learner groups. The analysis revealed a strong interaction effect between proficiency level and tone sequence (X^2^ = 1078.3, df = 47, *p* < 0.001). To further investigate this interaction, we performed separate analyses for each tone sequence across the three learner groups, all of which showed significant differences (*p* < 0.001). Additionally, post hoc multiple comparisons were conducted to determine whether accuracy rates for each tone sequence significantly varied among the three groups. Table 5 presents the results of these comparisons, displaying only estimate coefficients (Est) and *p*-values for brevity. As shown in Table 5, significant differences were found between advanced and beginner learners, as well as between beginner and intermediate learners. While most tone sequences also exhibited significant differences between advanced and intermediate learners, two sequences—T1−T2 and T4−T2—did not show statistically significant differences between these two groups (*p* = 0.321 and *p* = 0.58, respectively).

The statistical results above confirm that the three learner groups differed considerably in their production of the 16 tone sequences. To further investigate these differences, we conducted a binary logistic regression analysis to determine whether the production of the tone sequences varied within each learner group. The results revealed a highly significant main effect on accuracy rates across all three groups (Advanced: X^2^ = 387.5, df = 15, *p* < 0.001, Intermediate: X^2^ = 392.8, df = 15, *p* < 0.001, Beginner: X^2^ = 257.0, df = 15, *p* < 0.001). To identify which tone sequences exhibited significant differences, post hoc multiple comparisons within each learner group were performed across all possible sequence pairs within the 16 sequences, yielding a total of 105 pairwise comparisons. These comparisons provided a detailed assessment of how specific tone sequences were distinguished or confused within each learner group, offering insights into patterns of production difficulty. However, since displaying such a large number of comparisons is neither practical nor informative, we selected only the five easiest and five most difficult tone sequences (based on the rankings in Table 4). These sequences were used to assess which tone sequences learners found easier or more challenging to produce. The results are presented in Table 6, Table 7 and Table 8 below, organized by proficiency level from beginner to advanced learners.

Table 6 presents the results of post hoc multiple comparisons assessing differences in production accuracy among selected tone sequences within the beginner group. The table is divided into two sections: Easiest and hardest tone sequences, indicating which sequences were more or less challenging for learners to produce. The statistical results reveal clear distinctions in the production difficulty of different tone sequences for beginner learners. In the easiest tone sequences, several significant differences were observed, particularly in comparisons involving T1–T1, such as T1–T1 vs. T1–T3 (*p* < 0.001), T1–T1 vs. T2–T3 (*p* < 0.001), and T1–T1 vs. T2–T1 (*p* < 0.001). These results suggest that T1–T1 was the easiest sequence overall for the beginner group. In contrast, comparisons such as T3–T1 vs. T1–T3 (*p* = 0.732) and T3–T1 vs. T2–T3 (*p* = 0.464) did not reach statistical significance, indicating that these sequences posed a similar level of difficulty for beginners to produce. In the hardest tone sequences, fewer significant differences were observed. The contrast T1–T2 vs. T3–T2 (*p* = 0.001) was the only statistically significant comparison, suggesting that T3–T2 was the most challenging sequence to produce in the beginner group. However, most other comparisons, such as T4–T1 vs. T1–T2 (*p* = 0.998) and T4–T2 vs. T3–T2 (*p* = 0.978), did not reach significance, indicating that these sequences were produced with comparable difficulty. Notably, sequences ending in Tone 2, such as T4–T2, T3–T2, and T2–T2, were among the most challenging sequences to produce, as reflected in the lack of significant differences in many of the pairwise comparisons of each T2-final sequence (T4–T2, T3–T2, T2–T2) with the other tone sequences.

Table 7 presents the results of post hoc multiple comparisons evaluating differences in production accuracy among selected tone sequences within the intermediate learner group. In the easiest tone sequences, most comparisons did not yield significant differences, indicating that these sequences were perceived with similar accuracy levels. However, the contrast between T3-T1 and T1–T3 (*p* = 0.041) reached statistical significance, suggesting that intermediate learners produced these two sequences more accurately than the others. Among the hardest tone sequences, significant differences were observed, particularly in comparisons involving T4–T4 vs. T3–T2 (*p* = 0.002) and T4–T4 vs. T2–T2 (*p* = 0.001). These results indicate that T3–T2 and T2–T2 were among the most challenging sequences to produce for intermediate learners, as their accuracy rates were significantly lower when compared to T4–T4. However, other comparisons among sequences ending in Tone 2 did not reach statistical significance, indicating that these sequences were similarly difficult for intermediate learners to produce.

Table 8 presents the results of multiple comparisons for tone sequences produced by advanced learners. Among the easiest tone sequences, all comparisons yielded non-significant differences (*p* > 0.05), suggesting that advanced learners produced these sequences with similar accuracy levels. In contrast, several significant differences emerged among the hardest tone sequences. In particular, the contrasts T4–T4 vs. T4–T2 (*p* = 0.004) and T4–T4 vs. T2–T2 (*p* = 0.004) indicate that T4–T2 and T2–T2 were among the most challenging sequences to produce. Additionally, T3–T2 vs. T2–T2 (*p* = 0.002) suggests that T2–T2 was particularly difficult compared to T3–T2. However, some comparisons, such as T1–T2 vs. T4–T2 (*p* = 1.000) and T4–T2 vs. T2–T2 (*p* = 1.000), did not reach significance, indicating that these sequences were produced with comparable difficulty. Overall, the results indicate that even at the advanced proficiency level, tone sequences ending in Tone 2 remain particularly difficult to produce. This finding may have implications for targeted pronunciation training, which will be further discussed in Section 4.

**Figure 3:**
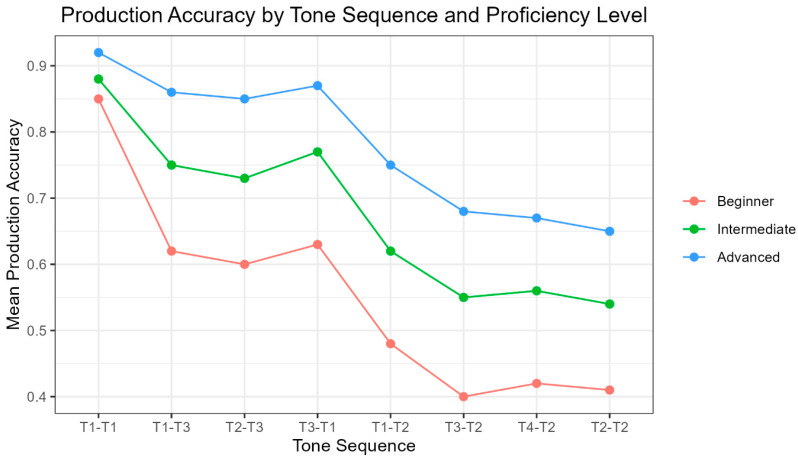
Mean production accuracy for different tone sequences across proficiency groups.

To improve the accessibility of the multiple-comparison results reported in Table 6, Table 7 and Table 8, we provide a summary that highlights the key patterns observed across proficiency levels. Figure 3 presents selected tone sequences representing the easiest, hardest, and intermediate levels of difficulty. Mean production accuracy increases with proficiency across all sequences. Advanced learners consistently outperformed intermediate and beginner learners. T1–T1 was produced with the highest accuracy, whereas sequences ending in Tone 2 (T1–T2, T3–T2, T4–T2, and T2–T2) showed the lowest accuracy. Sequences such as T1–T3 and T2–T3 fell between the easiest and hardest sequences across all groups.

### 3.3. Error Patterns

As demonstrated in Section 3.1, all sequences with T2 in the second syllable showed a significantly lower accuracy rate compared to other sequences. To provide a more detailed view of these patterns, we present error patterns for each learner group in Table 9, Table 10 and Table 11, offering insights into both correct and incorrect productions. In each table, the rows represent the target tone sequences, while the columns list the four most frequent productions in descending order. The fifth column consolidates all other productions. Numbers in brackets indicate response percentages. For instance, “T1–T1 (65.2)” in the first row signifies that T1–T1 was correctly produced 65.2% of the time, whereas “T1–T4 (21.1)” indicates that T1–T1 was misproduced as T1–T4 in 21.1% of cases.

Table 9 shows that beginner learners had an overall accuracy rate of 31.4% across the 16 target tone sequences. They produced a wider range of variant forms for the same sequences, with each target sequence yielding more than five different variants. Misproductions were particularly frequent when T2 and T4 appeared in the second syllable. For example, T3–T2 had the lowest accuracy rate (8.0%) and was often mistaken for T2–T3 nearly a third of the time (32.4%), T3-T1 in 20.5% of cases, and T1–T3 in 11.9%. The sequence with the second-lowest accuracy rate (11.9%), T2–T2, was frequently misread as T2–T3 (26.8%) and as T1–T3 (12.5%). T4–T2, which had the third-lowest accuracy rate (12.8%), was most commonly confused with T4–T3 (24.1%) and T1–T3 (18.5%). Finally, T1–T2, with the fourth-lowest accuracy rate (22.9%), was mispronounced as T1–T3 in 36.3% of cases. Additionally, T4 was sometimes produced as T1, though these errors occurred less frequently compared to the misproduction of T2 as T3. For instance, T1–T4 was most frequently confused with T1–T1 (36%), T3–T4 with T3-T1 (33.3%), and T4–T4 with T4–T1 (27.1%).

Table 10 shows that intermediate learners had an overall accuracy rate of 57.1% across the 16 target tone sequences, which was 25.7% higher than that of beginner learners (31.4%). They produced fewer variant forms than beginners for the same tone sequences, though each target sequence still elicited more than five different variants. Mispronunciations remained common, particularly when T2 and T4 appeared in the second syllable. For example, T2–T2 and T3–T2 had the lowest accuracy rate (31.8%). T2–T2 was most frequently mistaken for T2–T3 (51.8%) and, to a lesser extent, T3–T2 (5.1%). T3–T2 was confused with T2–T3 in 49.7% of cases and with T3-T1 in 11.9%. The sequence with the third-lowest accuracy rate, T4–T2 (45.2%), was most often misproduced as T4–T3 (35.4%) and T1–T3 (11.9%). Finally, T1–T2, which had the fourth-lowest accuracy rate (49.1%), was misread as T1–T3 in 36.6% of cases. As in the beginner group, T4 was sometimes confused with T1, though these errors were less frequent than the misproduction of T2 as T3. For instance, T4–T4 was most commonly mistaken for T4–T1 (40.5%), T3–T4 for T3-T1 (36.6%), and T1–T4 for T1–T1 (36.3%).

As shown in Table 11, advanced learners achieved an overall accuracy rate of 79.95% across the 16 target tone sequences, which was 22.85% higher than that of intermediate learners (57.1%). They produced fewer variant forms for the same tone sequences compared to intermediates—only two for T1–T1, three each for T2–T1, T3-T1, and T3–T4, and four each for T1–T4, T2–T2, T2–T3, T2-T4, T4–T2, T4–T3, and T4–T4—whereas intermediate learners exhibited more than five variants for each sequence. This reduction in production variation suggests that advanced learners made notable progress in correctly producing Mandarin tone sequences. Nevertheless, some persistent errors remained, particularly the tendency to confuse T2 with T3 and T4 with T1. The former, especially in the final syllable, was evident in the misproduction of T1–T2 as T1–T3 (39.3%). Other frequently observed confusions included T2–T2 misread as T2–T3 (45.5%), T4–T2 mistaken for T4–T3 (36.3%), and T3–T2 misproduced as T2–T3 (25.6%). This consistent mispronunciation of T2 as T3 highlights an ongoing challenge, even for advanced learners—a point that will be further explored in Section 4.

### 3.4. Accuracy Rates in Each Syllable

**Figure 4:**
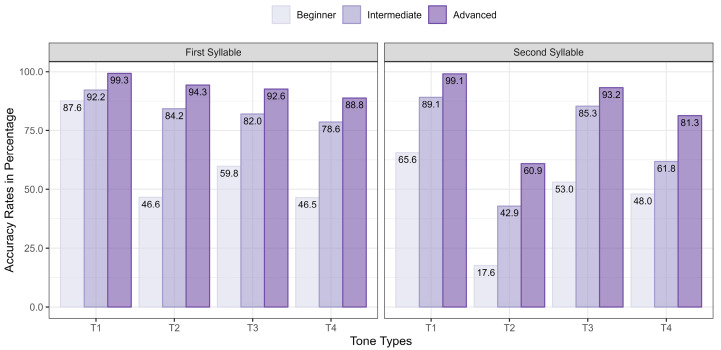
Accuracy rates for each tone in both the first and second syllables.

Figure 4 illustrates the accuracy rates for each tone in the first and second syllables across proficiency levels. Advanced learners consistently achieved the highest accuracy, followed by intermediate learners and then beginners. In the first syllable, both intermediate and advanced learners had accuracy rates exceeding 80% and 90%, respectively, for all tones except T4. Beginners, however, showed a noticeable gap compared to the other groups, except for T1. Their accuracy rate for T3 was 59.8%, while for T2 and T4, it dropped to 46.6% and 46.5%, respectively. In the second syllable, accuracy rates generally declined, with T2 showing particularly low accuracy. Advanced learners achieved a 60.9% accuracy rate for T2, whereas beginners’ accuracy dropped significantly to 17.6%. Intermediate learners’ accuracy fell between the two groups.

The binary logistic regression revealed a strong three-way interaction among proficiency level, tone sequence, and position (X^2^ = 2169.4, df = 23, *p* < 0.001). Separate analyses for each group indicated a strong two-way interaction between tone sequence and position (Beginner: X^2^ = 769.9, df = 7, *p* < 0.001, Intermediate: X^2^ = 736.1, df = 7, *p* < 0.001, Advanced: X^2^ = 616.2, df = 7, *p* < 0.001). According to the post hoc analysis, beginners showed the highest accuracy for T1 in the first syllable (*p* < 0.001). T3 was more accurate than T2 (*p* < 0.001), but there was no significant difference between T2 and T4 or between T3 and T4 (*p* > 0.1). In the second syllable, T1 was significantly more accurate than all other tones (*p* < 0.001), while T3 and T4 had similar accuracy levels (*p* > 0.1). T2 had the lowest accuracy (*p* < 0.001). Intermediate and advanced learners exhibited similar patterns. In the first syllable, T1 was significantly more accurate than all other tones (*p* < 0.001). T2 was more accurate than T4 (*p* < 0.001) but did not differ significantly from T3 (*p* > 0.1), while T3 and T4 showed no significant difference (*p* > 0.1). In the second syllable, T1 again had the highest accuracy (*p* < 0.001). Among intermediate learners, T1 and T3 did not differ significantly (*p* > 0.1). In both groups, T2 was markedly less accurate than T3 and T4 (*p* < 0.001), with T3 being more accurate than T4 (*p* < 0.001).

## 4. Discussion

In this study, we observed the overall tendencies and error patterns of 16 tone sequences in Mandarin disyllabic words produced by Korean learners of Mandarin at beginner, intermediate, and advanced levels. Our results revealed that learners made significant progress in almost every tone sequence as their proficiency improved and that the learner groups exhibited similar rankings of the tone sequences, with differences mainly in accuracy rates. The error patterns remained consistent across groups, with only slight variations. Regardless of proficiency level, T1–T1 and T3–T1 were the easiest, while sequences ending with T2 were the most challenging. The error patterns showed that T2 and T4 were frequently mispronounced as T3 and T1, respectively. These consistent patterns underscore the influence of Korean learners’ L1 prosodic systems on their production of Mandarin tone sequences.

Returning to the research questions made in this study, our results confirmed Question 1, which stated that Korean learners would have an advantage when producing tone sequences similar to Korean tonal patterns. Sequences such as T1–T1 and T3–T1, labeled as H-H and LH-H, consistently ranked among the most accurately produced across all proficiency levels. These findings highlight the beneficial effects of positive transfer from L1 to L2, facilitating the acquisition of Mandarin tone sequences. Even learners from non-tonal L1 backgrounds may find tone sequences easier to acquire if they resemble those in their native language. Our results, therefore, align with previous research suggesting that the degree of correspondence between L1 prosodic patterns and L2 tone pairs influences acquisition patterns [25,28] and that linguistic similarities between two languages positively impact foreign language acquisition [29].

This study also validated Question 2: Korean learners struggled to produce tone sequences that do not resemble Korean tonal patterns. Tone pairs ending in T2 were particularly challenging and warrant further discussion. As shown in Table 5, no significant differences were found between intermediate and advanced learners for the sequences T1–T2 and T4–T2, indicating that sequences ending in T2 remained difficult even at higher proficiency levels. Furthermore, as illustrated in Figure 4, even advanced learners achieved only a 60.9% accuracy rate for T2 in the second syllable—significantly lower than the average accuracy rate of over 90% for other tones. This difficulty likely stems from the fact that Mandarin T2 has no equivalent in Korean prosody. As a result, Korean learners struggled with tone sequences ending in T2 regardless of their proficiency level. Rather than correctly targeting the mid-pitch onset of T2, they often lowered its initial pitch to align with the low pitch of T3, creating a pattern similar to the LH contour in Korean. Consequently, T1–T2, T2–T2, and T4–T2 were frequently mispronounced as T1–T3, T2–T3, and T4–T3, respectively (see Table 9, Table 10 and Table 11). This validation of Question 2 underscores the study’s implications for understanding how learners map their L1 prosodic patterns onto L2 tone sequences.

In contrast, pronouncing T2 was less challenging in the first syllable compared to the second. This is similar to findings of learners from English background as in Hao [53]. What factors contributed to this difference? We speculate that the increased difficulty of T2 in the second syllable likely stemmed from the influence of preceding tones, a phenomenon known as carry-over effects due to tonal coarticulation [47]. This effect makes it harder to exert precise control for accurate tone production because learners have not mastered tonal coarticulation patterns to the same extent as native speakers. Even advanced learners struggled with pronouncing T2 accurately in the second syllable, achieving only about 61% accuracy. The challenge of producing T2 in the second syllable persisted despite learners’ development of proficiency. Korean learners, however, did not face the same difficulty with T2 in the first syllable. While beginner learners had an accuracy rate of 46.6%, this rate significantly increased with proficiency: intermediate learners achieved 84.2%, and advanced learners reached 94.3%. These findings suggest that while T2 in the first syllable was initially challenging, Korean learners gradually overcame this difficulty as their proficiency improved.

Question 2 also asked if T4–T1 (HL-H) would be particularly challenging for Korean learners. This was partially confirmed: tone pairs beginning with T4, including T4–T1 and T4–T4, were ranked among the most difficult, similar to the pairs ending with T2. Beginners had an accuracy rate of 22.9% for T4–T1, similar to their rate for T1–T2 (22.9%). However, as learners advanced, their accuracy for T4–T1 improved significantly to 75.9%. These results indicate that while T4–T1 was initially as challenging as T1–T2, Korean learners overcame this difficulty as their proficiency increased. We speculate that Korean learners may find acquiring a falling tone easier than a mid tone. This might be due to the falling tone’s similarity to existing tonal patterns in Korean, although it typically appears only sentence-finally. In contrast, a mid tone has no equivalent within a sentence in Korean, making it harder to acquire.

Beyond the aforementioned prosodic transfer and carry-over effects, the persistent difficulty with T2 sequences can also be understood from a neurolinguistic perspective. As outlined in the Introduction, the motor theory of speech perception suggests that learners rely on the neural simulation of familiar motor gestures to process and reproduce tones [30,31]. While Korean learners can effectively produce existing motor routines for sequences such as T1–T1 and T3–T1, Mandarin T2 lacks a corresponding motor template in the Korean prosodic system. This absence of motor equivalence leads to unstable motor simulation and thus hinders accurate reproduction of T2, even at advanced proficiency levels.

One may wonder why T2 in word-initial position is improved with experience. With increased exposure and practice, sensorimotor representations for rising pitch patterns gradually become more stable and automatized. In other words, learners begin to form new motor routines (or partial “motor templates”) specific to Mandarin rising tones, supported by strengthened auditory–motor coupling through neural plasticity. This process is consistent with findings showing that training and experience enhance cortical activation and connectivity between auditory and premotor regions involved in tone production and perception [54,55]. Over time, such experience-dependent reorganization enables learners to anticipate and execute rising F0 movements more precisely when the tone occurs in isolation or at the beginning of a sequence. However, in word-final position where carry-over interference from preceding tones is present, learners have particularly difficulty producing accurate sequences ending in T2.

It has been well attested that lexical frequency and familiarity can influence phonetic accuracy, as widely demonstrated in phonetic and L2 research. For example, Llompart [56] shows that learners’ ability to encode and reject nonwords involving difficult L2 vowel contrasts is shaped by both lexical factors (e.g., frequency, neighborhood density) and acoustic properties. Importantly, these factors affect different vowels (/æ/and/ε/in that study) asymmetrically, suggesting that less robust, non-native vowels may rely more heavily on lexical structure and L2 experience than their more stable counterparts. Similarly, Beaman and Tomaschek [57] demonstrate that sound change across the lifespan is shaped by interactions among phonetic environment, lexical frequency, and social identity, with frequency-related effects modulating the degree of phonetic contrast merger in a community- and speaker-specific manner. In the present study, however, lexical frequency was not treated as an independent variable, as stimulus selection prioritized phonological and distributional constraints relevant to tone-sequence production. Most items were drawn from graded vocabulary lists: high-frequency words constituted 220 of the 224 items, with only four low-frequency words included as exceptions where the required constraints could not be satisfied using high-frequency items alone. These items were evenly distributed across tone-sequence conditions; therefore, lexical frequency is unlikely to have systematically biased the observed difficulty patterns. Nonetheless, in line with previous findings, we acknowledge that lexical familiarity may interact with tone accuracy, and we now explicitly note this as a limitation and an important direction for future research.

The results of this study provide pedagogical guidance on how tone sequences may be taught in L2 pronunciation instruction. Despite learners’ difficulties with the production of T2, the accuracy rate of T2–T3 ranked third in both of the intermediate and advanced groups. Notably, this sequence is segmentally identical to T3–T2, differing only in tonal order, yet T3–T2 was among the most challenging sequences to produce, ranking second in difficulty for the intermediate group and fourth for the advanced group. This asymmetry highlights the role of tonal sequencing and draws attention to the T3 sandhi rule, raising the question of why even intermediate learners were able to produce T2–T3 with relatively high accuracy. T2–T3 is known to be typically introduced through explicit instruction and reinforced through sustained corrective feedback in Mandarin classrooms, leading Korean learners—regardless of overall proficiency—to develop a robust command of this sequence. This pedagogical emphasis may also facilitate learners’ ability to identify the surface realization of T2 as a rising contour that passes through a mid-pitch region. Because Korean prosody lacks a mid tone, learners may initially struggle to perceive or intentionally target mid-level pitch targets. However, explicit instruction appears to provide a clear auditory reference point for this unfamiliar pitch category. Taken together, these findings suggest that the high accuracy of T2–T3 reflects sustained instructional reinforcement, and that comparable proficiency in other T2-containing sequences may be achievable with similarly targeted instruction and feedback.

## 5. Conclusions

To summarize, we conclude by highlighting the linguistic and neurolinguistic implications of this study and by outlining a direction for future research. By comparing the prosodic structures of Mandarin and Korean, we explained why Korean learners encountered persistent difficulty with certain disyllabic tone sequences—especially T1–T2 and T4–T1—as a result of negative transfer. Beyond prosodic mismatch, our findings align with neurolinguistic evidence that tones lacking L1 motor counterparts suffer from both the absence of motor templates and weakened auditory encoding. These constraints provide a principled explanation for the long-lasting challenge of producing T2 sequences across proficiency levels. In this sense, the study contributes to a broader understanding of how L1-specific motor repertoires and auditory salience jointly shape the acquisition of L2 tones. To extend this line of inquiry, future research could compare learners from different L1 backgrounds to examine how prosodic differences interact with neurolinguistic constraints. For instance, comparing Japanese and Korean learners—both from non-tonal L1 backgrounds but with distinct intonational repertoires—would clarify whether the persistent difficulty with Mandarin T2 reflects universal neurolinguistic limitations or L1-specific influences.

## Figures and Tables

**Table 1:** Four lexical tones of Mandarin in isolation.

Tone	Pitch Value	Pinyin	Meaning
Tone 1 (T1)	55	dā	build
Tone 2 (T2)	35	dá	reach
Tone 3 (T3)	214	dǎ	hit
Tone 4 (T4)	51	dà	big

Note. Diacritics above vowel letters in Column 3 indicate similar shapes to their respective pitch contours.

**Table 2:** The 16 disyllabic tone sequences.

	__T1	__T2	__T3	__T4
T1__	kā.fēi (coffee)	ā.yí (aunt)	gāng.bǐ (pen)	tiān.qì (weather)
T2__	yá.gāo (toothpaste)	hé.gé (qualify)	jí.tǐ (group)	wán.jù (toy)
T3__	kǎo.yā (roasted duck)	yǔ.yán (language)	lǎo.hǔ (tiger)	kě.kào (reliable)
T4__	dà.mā (aunt)	yuè.dú (reading)	dà.mǐ (rice)	gù.kè (customer)

Note. Syllable boundaries are marked by dots.

**Table 3:** Mandrin disyllabic tone sequences and corresponding tonal patterns in Korean.

Tone Sequence	Mandarin	Korean	Tone Sequence	Mandarin	Korean
T1T1	HH	HH	T3T1	LH	LHH
T1T2	HMH		T3T2	LMH	
T1T3	HL	HLH	T3T3	MHL	
T1T4	HHL	HHL	T3T4	LHL	LHHL
T2T1	MHH		T4T1	HLH	HLH
T2T2	MHMH		T4T2	HLML	
T2T3	MHL		T4T3	HLL	HLLH
T2T4	MHHL		T4T4	HLHL	HLHL

**Table 4:** Rankings for the 16 tone sequences in each learner group.

Ranking	Beginner		Intermediate		Advanced	
	Tone	Accuracy	Tone	Accuracy	Tone	Accuracy
1	T1−T1	65.2%	T1−T1	87.5%	T1−T1	99.7%
2	T3−T1	50.9%	T3−T1	86.6%	T3−T1	99.4%
3	T1−T3	42.6%	T2−T3	83.3%	T2−T3	95.2%
4	T2−T3	40.5%	T3−T3	82.7%	T3−T3	93.8%
5	T2−T1	36.6%	T1−T3	70.5%	T1−T3	91.1%
6	T1−T4	36.0%	T2−T1	68.5%	T2−T1	86.6%
7	T3−T3	34.2%	T4−T3	62.8%	T4−T3	86.3%
8	T3−T4	32.4%	T1−T4	57.1%	T3−T4	84.5%
9	T4−T3	30.4%	T3−T4	56.8%	T1−T4	79.2%
10	T2−T4	25.6%	T2−T4	55.1%	T2−T4	76.8%
11	T4−T4	23.2%	T4−T1	54.8%	T4−T1	75.9%
12	T4−T1	22.9%	T4−T4	53.6%	T4−T4	73.2%
13	T1−T2	22.9%	T1−T2	49.1%	T3−T2	69.9%
14	T4−T2	12.8%	T4−T2	45.2%	T1−T2	59.2%
15	T2−T2	11.9%	T3−T2	31.8%	T4−T2	54.8%
16	T3−T2	8.0%	T2−T2	31.8%	T2−T2	53.6%

**Table 5:** Results of post hoc multiple comparisons for each tone sequence across the three learner groups.

Tone Sequence	Advanced-Beginner	Advanced-Intermediate	Beginner-Intermediate
T1−T1	Est: 4.88, *p* < 0.001	Est: 3.403, *p* = 0.003	Est: −1.476, *p* < 0.001
T1−T2	Est: 1.296, *p* < 0.001	Est: 0.355, *p* = 0.321	Est: −0.941, *p* = 0.001
T1−T3	Est: 3.766, *p* < 0.001	Est: 1.515, *p* < 0.001	Est: −2.251, *p* < 0.001
T1−T4	Est: 2.643, *p* < 0.001	Est: 2.018, *p* < 0.001	Est: −0.626, *p* = 0.03
T2−T1	Est: 2.85, *p* < 0.001	Est: 1.29, *p* < 0.001	Est: −1.560, *p* < 0.001
T2−T2	Est: 1.966, *p* < 0.001	Est: 0.771, *p* = 0.003	Est: −1.195, *p* < 0.001
T2−T3	Est: 4.165, *p* < 0.001	Est: 1.248, *p* = 0.014	Est: −2.916, *p* < 0.001
T2−T4	Est: 2.352, *p* < 0.001	Est: 1.054, *p* < 0.001	Est: −1.298, *p* < 0.001
T3−T1	Est: 5.052, *p* < 0.001	Est: 2.34, *p* = 0.007	Est: −2.712, *p* < 0.001
T3−T2	Est: 4.405, *p* < 0.001	Est: 2.269, *p* < 0.001	Est: −2.136, *p* < 0.001
T3−T3	Est: 4.42, *p* < 0.001	Est: 1.185, *p* = 0.014	Est: −3.236, *p* < 0.001
T3−T4	Est: 3.24, *p* < 0.001	Est: 2.199, *p* < 0.001	Est: −1.041, *p* < 0.001
T4−T1	Est: 3.131, *p* < 0.001	Est: 1.402, *p* < 0.001	Est: −1.729, *p* < 0.001
T4−T2	Est: 1.883, *p* < 0.001	Est: 0.235, *p* = 0.58	Est: −1.648, *p* < 0.001
T4−T3	Est: 3.156, *p* < 0.001	Est: 1.522, *p* < 0.001	Est: −1.634, *p* < 0.001
T4−T4	Est: 2.494, *p* < 0.001	Est: 1.077, *p* < 0.001	Est: −1.418, *p* < 0.001

Note. *p*-values in gray mean no significant differences.

**Table 6:** Results of multiple comparisons for tone sequences in the beginner group.

	Contrast	Estimate	SE	z-Value	*p*-Value
Easiest	T1–T1 vs. T1–T3	0.621	0.244	2.54	0.442
	T1–T1 vs. T1–T3	1.138	0.246	4.624	<0.001
	T1–T1 vs. T2–T3	1.226	0.247	4.965	<0.001
	T1–T1 vs. T2–T1	1.316	0.248	5.306	<0.001
	T3–T1vs. T1–T3	−0.517	0.241	−2.151	0.732
	T3–T1 vs. T2–T3	−0.606	0.241	−2.51	0.464
	T3–T1 vs. T2–T1	−0.695	0.242	−2.869	0.231
	T1–T3 vs. T2–T3	0.088	0.242	0.364	1
	T1–T3 vs. T2–T1	0.178	0.243	0.73	1
	T2–T3 vs. T2–T1	−0.089	0.244	−0.366	1
Hardest	T4–T1 vs. T1–T2	0.320	0.267	1.197	0.998
	T4–T1 vs. T4–T2	0.563	0.297	1.894	0.879
	T4–T1 vs. T2–T2	−0.105	0.323	−0.324	1
	T4–T1 vs. T3–T2	−1.103	0.332	−3.32	0.069
	T1–T2 vs. T4–T2	0.883	0.29	3.043	0.151
	T1–T2 vs. T2–T2	0.987	0.296	3.338	0.066
	T1–T2 vs. T3–T2	1.423	0.326	4.367	0.001
	T4–T2 vs. T2–T2	−0.105	0.323	−0.324	1
	T4–T2 vs. T3–T2	−0.540	0.351	−1.539	0.978
	T2–T2 vs. T3–T2	0.436	0.356	1.225	0.998

Note. Estimate represents the estimated difference in production accuracy between the two sequences. SE is the standard error of the estimate. *p*-values in gray mean significant differences.

**Table 7:** Results of multiple comparisons for tone sequences in the intermediate group.

	Contrast	Estimate	SE	z-Value	*p*-Value
Easiest	T1–T1 vs. T1–T3	−0.430	0.334	−1.288	0.996
	T1–T1 vs. T2–T3	−0.095	0.313	−0.303	1
	T1–T1 vs. T3–T3	0.089	0.304	0.293	1
	T1–T1 vs. T1–T3	0.659	0.284	2.324	0.605
	T3–T1 vs. T2–T3	−0.335	0.339	−0.989	1
	T3–T1 vs. T3–T3	0.519	0.331	1.57	0.974
	T3–T1 vs. T1–T3	−1.089	0.312	−3.487	0.041
	T2–T3 vs. T3–T3	0.184	0.309	0.596	1
	T2–T3 vs. T1–T3	−0.754	0.289	−2.606	0.394
	T3–T3 vs. T1–T3	−0.570	0.279	−2.042	0.802
Hardest	T4–T4 vs. T1–T2	−0.437	0.228	−1.916	0.869
	T4–T4 vs. T4–T2	−0.507	0.229	−2.219	0.684
	T4–T4 vs. T3–T2	−0.997	0.234	−4.261	0.002
	T4–T4 vs. T2–T2	−1.024	0.234	−4.368	0.001
	T1–T2 vs. T4–T2	0.070	0.228	0.309	1
	T1–T2 vs. T3–T2	0.560	0.233	2.402	0.546
	T1–T2 vs. T2–T2	0.587	0.234	2.514	0.461
	T4–T2 vs. T3–T2	−0.489	0.233	−2.097	0.767
	T4–T2 vs. T2–T2	−0.517	0.234	−2.21	0.690
	T3–T2 vs. T2–T2	−0.028	0.239	−0.115	1

Note. *p*-values in gray mean significant differences.

**Table 8:** Results of multiple comparisons for tone sequences in the advanced group.

	Contrast	Estimate	SE	z-Value	*p*-Value
Easiest	T1–T1 vs. T1–T3	0.697	1.216	0.573	1
	T1–T1 vs. T2–T3	2.147	1.053	2.039	0.804
	T1–T1 vs. T3–T3	2.398	1.041	2.302	0.622
	T1–T1 vs. T1–T3	2.763	1.028	2.688	0.338
	T3–T1 vs. T2–T3	−1.450	0.8	−1.814	0.912
	T3–T1 vs. T3–T3	1.701	0.784	2.169	0.720
	T3–T1 vs. T1–T3	−2.066	0.766	−2.698	0.331
	T2–T3 vs. T3–T3	0.250	0.494	0.507	1
	T2–T3 vs. T1–T3	−0.616	0.464	−1.327	0.995
	T3–T3 vs. T1–T3	−0.365	0.437	−0.836	1
Hardest	T4–T4 vs. T3–T2	−0.169	0.27	−0.627	1
	T4–T4 vs. T1–T2	−0.851	0.259	−3.282	0.078
	T4–T4 vs. T4–T2	−1.076	0.26	−4.143	0.004
	T4–T4 vs. T2–T2	−1.076	0.26	−4.143	0.004
	T3–T2 vs. T1–T2	−0.681	0.255	−2.669	0.351
	T3–T2 vs. T4–T2	0.907	0.256	3.538	0.035
	T3–T2 vs. T2–T2	−1.086	0.255	−4.259	0.002
	T1–T2 vs. T4–T2	0.225	0.242	0.929	1
	T1–T2 vs. T2–T2	0.404	0.241	1.68	0.952
	T4–T2 vs. T2–T2	−0.179	0.24	−0.745	1

Note. *p*-values in gray mean significant differences.

**Table 9:** Tone production confusion matrix for beginner learners.

Tone Pair	1 (%)	2 (%)	3 (%)	4 (%)	5 (%)
T1–T1	1-1 (65.2)	1-4 (21.1)	1-3 (6.8)	3-1 (3.3)	(3.6)
T1–T2	1-3 (36.3)	1-2 (22.9)	1-1 (17.9)	1-4 (7.1)	(15.8)
T1–T3	1-3 (42.6)	1-2 (26.2)	1-1 (10.4)	1-4 (8.6)	(12.2)
T1–T4	1-4 (36.0)	1-1 (36.0)	1-3 (12.5)	3-1 (4.8)	(10.7)
T2–T1	2-1 (36.6)	3-1 (18.5)	3-4 (11.9)	1-4 (9.2)	(23.8)
T2–T2	2-3 (26.8)	1-3 (12.5)	2-2 (11.9)	1-1 (11.6)	(38.0)
T2–T3	2-3 (40.5)	1-3 (17.3)	1-2 (9.5)	1-4 (8.6)	(24.1)
T2–T4	2-4 (25.6)	1-4 (18.5)	2-1 (14.3)	3-4 (14.0)	(27.6)
T3–T1	3-1 (50.9)	3-4 (17.3)	1-1 (11.3)	1-3 (9.5)	(11.0)
T3–T2	2-3 (32.4)	3-1 (20.5)	1-3 (11.9)	1-4 (8.6)	(26.6)
T3–T3	2-3 (34.2)	1-3 (10.1)	1-4 (8.6)	3-1 (8.6)	(38.5)
T3–T4	3-1 (33.3)	3-4 (32.4)	1-4 (13.7)	1-3 (8.6)	(12.0)
T4–T1	1-1 (33.9)	4-1 (22.9)	1-4 (16.7)	4-3 (5.7)	(20.8)
T4–T2	4-3 (24.1)	1-3 (18.5)	4-2 (12.8)	4-1 (11.3)	(33.3)
T4–T3	4-3 (30.4)	1-3 (28.0)	1-2 (10.4)	4-4 (6.5)	(24.7)
T4–T4	4-1 (27.1)	4-4 (23.2)	1-1 (15.2)	1-4 (13.1)	(21.4)

Note. Cells in gray mean wrongly produced tone sequences with a higher rate than the correctly produced ones.

**Table 10:** Tone production confusion matrix for intermediate speakers.

Tone Pair	1 (%)	2 (%)	3 (%)	4 (%)	5 (%)
T1–T1	1-1 (87.5)	1-4 (9.5)	3-1 (1.2)	1-3 (0.6)	(1.2)
T1–T2	1-2 (49.1)	1-3 (36.6)	4-3 (8.0)	1-1 (3.6)	(2.7)
T1–T3	1-3 (70.5)	1-2 (13.1)	4-3 (9.5)	1-1 (2.7)	(4.2)
T1–T4	1-4 (57.1)	1-1 (36.3)	1-3 (1.2)	3-1 (1.2)	(4.2)
T2–T1	2-1 (68.5)	3-1 (17.6)	2-4 (6.8)	2-3 (4.2)	(2.9)
T2–T2	2-3 (51.8)	2-2 (31.8)	3-2 (5.1)	2-1 (4.8)	(6.5)
T2–T3	2-3 (83.3)	1-3 (6.8)	3-2 (4.8)	2-2 (2.1)	(3.0)
T2–T4	2-4 (55.1)	2-1 (25.6)	3-4 (11.6)	1-4 (4.8)	(2.9)
T3–T1	3-1 (86.6)	3-4 (7.7)	1-1 (2.7)	2-3 (2.4)	(0.6)
T3–T2	2-3 (49.7)	3-2 (31.8)	3-1 (11.9)	2-2 (2.7)	(3.9)
T3–T3	2-3 (82.7)	3-1 (4.8)	1-3 (4.2)	3-2 (3.3)	(5.0)
T3–T4	3-4 (56.8)	3-1 (36.6)	1-4 (3.0)	1-1 (1.5)	(2.1)
T4–T1	4-1 (54.8)	1-1 (33.3)	4-4 (5.7)	1-4 (3.9)	(2.3)
T4–T2	4-2 (45.2)	4-3 (35.4)	1-3 (11.9)	1-2 (4.8)	(2.7)
T4–T3	4-3 (62.8)	1-3 (16.1)	4-2 (15.2)	2-3 (2.1)	(3.8)
T4–T4	4-4 (53.6)	4-1 (40.5)	1-1 (3.3)	1-4 (2.1)	(0.5)

Note. Cells in gray mean wrongly produced tone sequences with a higher rate than the correctly produced ones.

**Table 11:** Tone production confusion matrix for advanced learners.

Tone Pair	1 (%)	2 (%)	3 (%)	4 (%)	5 (%)
T1–T1	1-1 (99.7)	1-4 (0.3)			
T1–T2	1-2 (59.2)	1-3 (39.3)	4-3 (0.9)	1-1 (0.3)	(0.3)
T1–T3	1-3 (91.1)	1-2 (7.7)	4-3 (0.6)	1-1 (0.3)	(0.3)
T1–T4	1-4 (79.2)	1-1 (20.2)	2-4 (0.3)	3-4 (0.3)	
T2–T1	2-1 (86.6)	3-1 (12.8)	2-3 (0.6)		
T2–T2	2-2 (53.6)	2-3 (45.5)	2-1 (0.6)	4-2 (0.3)	
T2–T3	2-3 (95.2)	2-2 (4.2)	1-3 (0.3)	3-2 (0.3)	
T2–T4	2-4 (76.8)	2-1 (14.3)	3-4 (6.3)	3-1 (2.6)	
T3–T1	3-1 (99.4)	3-4 (0.3)	1-1 (0.3)		
T3–T2	3-2 (69.9)	2-3 (25.6)	2-2 (1.5)	3-1 (1.5)	(1.5)
T3–T3	2-3 (93.8)	2-2 (4.2)	3-2 (0.9)	4-3 (0.6)	(0.5)
T3–T4	3-4 (84.5)	3-1 (14.9)	2-3 (0.6)		
T4–T1	4-1 (75.9)	1-1 (21.4)	1-4 (1.5)	4-4 (0.3)	(0.9)
T4–T2	4-2 (54.8)	4-3 (36.3)	1-4 (4.8)	1-2 (4.1)	
T4–T3	4-3 (86.3)	4-2 (7.4)	1-3 (4.8)	1-2 (1.5)	
T4–T4	4-4 (73.2)	4-1 (20.8)	1-4 (4.8)	1-1 (1.2)	

## Data Availability

The data in this study will be available on request from the corresponding author due to privacy concerns.
